# CD8^+^ T-Cell Exhaustion Phenotype in Chronic Hepatitis C Virus Infection Is Associated With Epitope Sequence Variation

**DOI:** 10.3389/fimmu.2022.832206

**Published:** 2022-03-21

**Authors:** Sylwia Osuch, Tomasz Laskus, Karol Perlejewski, Hanna Berak, Iwona Bukowska-Ośko, Agnieszka Pollak, Magdalena Zielenkiewicz, Marek Radkowski, Kamila Caraballo Cortés

**Affiliations:** ^1^ Department of Immunopathology of Infectious and Parasitic Diseases, Medical University of Warsaw, Warsaw, Poland; ^2^ Department of Adult Infectious Diseases, Medical University of Warsaw, Warsaw, Poland; ^3^ Outpatient Clinic, Warsaw Hospital for Infectious Diseases, Warsaw, Poland; ^4^ Department of Human Genetics, Medical University of Warsaw, Warsaw, Poland; ^5^ Institute of Mathematics, University of Warsaw, Warsaw, Poland

**Keywords:** hepatitis C virus, T-cell exhaustion, PD-1, Tim-3, epitope sequence

## Abstract

**Background and Aims:**

During chronic hepatitis C virus (HCV) infection, CD8^+^ T-cells become functionally exhausted, undergoing progressive phenotypic changes, i.e., overexpression of “inhibitory” molecules such as PD-1 (programmed cell death protein 1) and/or Tim-3 (T-cell immunoglobulin and mucin domain-containing molecule-3). The extreme intrahost genetic diversity of HCV is a major mechanism of immune system evasion, facilitating epitope escape. The aim of the present study was to determine whether T-cell exhaustion phenotype in chronic HCV infection is related to the sequence repertoire of NS3 viral immunodominant epitopes.

**Methods:**

The study population was ninety prospective patients with chronic HCV genotype 1b infection. Populations of peripheral blood CD8^+^ T-cells expressing PD-1/Tim-3 were assessed by multiparametric flow cytometry, including HCV-specific T-cells after magnetic-based enrichment using MHC-pentamer. Autologous epitope sequences were inferred from next-generation sequencing. The correction of sequencing errors and genetic variants reconstruction was performed using Quasirecomb.

**Results:**

There was an interplay between the analyzed epitopes sequences and exhaustion phenotype of CD8^+^ T-cells. A predominance of NS3_1406_ epitope sequence, representing neither prototype KLSGLGLNAV nor cross-reactive variants (KLSSLGLNAV, KLSGLGINAV or KLSALGLNAV), was associated with higher percentage of HCV-specific CD8^+^PD-1^+^Tim-3^+^ T-cells, P=0.0102. Variability (at least two variants) of NS3_1406_ epitope sequence was associated with increased frequencies of global CD8^+^PD-1^+^Tim-3^+^ T-cells (P=0.0197) and lower frequencies of CD8^+^PD-1^−^Tim-3^−^ T-cells (P=0.0079). In contrast, infection with NS3_1073_ dominant variant epitope (other than prototype CVNGVCWTV) was associated with lower frequency of global CD8^+^PD-1^+^Tim-3^+^ T-cells (P=0.0054).

**Conclusions:**

Our results indicate that PD-1/Tim-3 receptor expression is largely determined by viral epitope sequence and is evident for both HCV-specific and global CD8^+^ T-cells, pointing to the importance of evaluating autologous viral epitope sequences in the investigation of CD8^+^ T-cell exhaustion in HCV infection.

## Introduction

Adaptive immune responses play a critical role in the clinical course of infection with hepatitis C virus (HCV) (1). Vigorous and polyfunctional cellular responses are indispensable for rapid viral load reduction and spontaneous recovery from HCV infection, which is observed in about 20%-50% of patients with acute hepatitis C (2–5). Previous studies have shown that CD8^+^ T-cell responses targeting multiple epitopes, in both structural and nonstructural viral proteins, are associated with viral clearance (5), while only a narrow set of epitopes are targeted in chronic infection (6, 7).

The extreme intrahost genetic diversity of HCV is the result of fast replication and high error rate of viral replicase (RNA-dependent-RNA polymerase) (8). It is manifested by the *quasispecies* phenomenon, which is the concomitant presence of closely related, but not identical genetic variants within an infected host, facilitating adaptive dynamics of the virus (8). This feature is postulated to be a major mechanism of immune system evasion, because of the increased probability of positive selection of escape variants within immune epitopes under immune pressure of the host (9–11). The heterogeneity of HCV epitopes may have direct clinical implications, including the development of chronic infection (11).

Previous studies have shown that there is an interplay between the strength of T-cell response in HCV infection and epitope escape; this may determine the outcome of acute infection, in particular the positive selection of mutations, which induce only a weak and thus potentially insufficient CD8^+^ T-cell response (12, 13). Evidence for CD8^+^ T-cell-mediated pressure was found both in the envelope and non-envelope proteins, including non-structural protein 3 (NS3) (14–16). NS3-derived antigens from HCV genotype 1B are presented in the HLA (human leucocyte antigen) context and contain many well defined CD4^+^ and CD8^+^ T cell epitopes, which are considered to be the most immunogenic among all HCV antigens (17).

Viral escape mutations typically occur within the first six months of infection in approximately 50% of the CD8^+^ T-cell-targeted epitopes (18, 19). In contrast, escape mutations during chronic infection are rare, which is likely due to the weak T-cell-mediated selection pressure (18). Escape mutations may be located at various positions within virus-specific CD8^+^ T-cell epitopes: HLA class I binding anchor, T-cell receptor contact site or the flanking region (20, 21). Amino acid substitutions may lead to altered proteasomal cleavage, including the loss of the original epitope (15, 22), impaired binding to the MHC molecule (19, 23), or compromised TCR recognition of mutated peptide–MHC complex (19).

In contrast to acute resolving infection, the quality of T-cell responses significantly deteriorates during chronic antigen stimulation, including progressive negative changes of their phenotype, function, and both epigenetic and transcriptional profile (24, 25). While both CD8^+^ and CD4^+^ HCV-specific T-cells may be present in liver tissue and peripheral blood, they are unable to clear the infection in most patients and do not prevent reinfection with HCV due to their functional exhaustion (1). T-cell exhaustion manifests itself as impairment of antiviral effector functions of antigen-specific T-cells: decline in the effector cytokines production, impaired elimination of infected cells, and decrease in the proliferative potential after antigen exposure *in vitro* (26–28). It is the persistent antigen exposure which is believed to be the major factor promoting T-cell exhaustion, but CD4^+^ T-cells deletion, activation of regulatory T-cells and increased anti-inflammatory cytokine (e.g. IL-10) production contribute as well (29, 30).

Phenotypic hallmarks of T-cell exhaustion are increased expression of “inhibitory” molecules, among them PD-1 (programmed cell death protein 1) and Tim-3 (T-cell immunoglobulin and mucin domain-containing molecule-3) on global and antigen-specific T-cells, which deliver negative signals precluding cell activation after antigen exposure (31).

PD-1/PD-L1 (programmed cell death protein 1 receptor/programmed cell death protein 1 ligand) inhibitory molecules are part of a regulatory pathway that has been reported to inhibit the virus-specific CD8^+^ cell function in lymphocytic choriomeningitis virus (LCMV) infection (32, 33). Engagement of the PD-1 receptor and its ligand inhibits cell cycle and synthesis of effector cytokines (32, 33). Previous studies performed both in acute and in chronic HCV infections indicated that PD-1 is expressed by HCV-specific CD8^+^ and CD4^+^ T-cells and that the blocking of the PD-1/PD-L1 pathway by anti-PD-L1 antibodies can improve proliferation of HCV-specific CD8^+^ cells (27, 28).

Exhausted T-cells do not always express PD-1, and the blocking of the PD-1/PD-L1 signaling pathway does not necessarily reconstitute Th1/Tc1 cytolytic function, suggesting that other inhibitory molecules may contribute to the exhaustion associated with chronic viral infections (34–36). One such molecule is Tim-3. Increased frequencies of Tim-3-expressing CD4^+^ and CD8^+^ T-cells have been observed in chronic HCV infection and were particularly high on HCV-specific CD8^+^ T-cells (37). Tim-3 expression correlates with a dysfunctional phenotype and reduced Th1/Tc1 cytokine production, but not with viral load. Blocking the Tim-3/Tim-3L interaction *in vitro* enhanced T-cell proliferation and cytolytic function in response to HCV antigens (36, 37). A single expression of PD-1 or other co-inhibitory receptors does not necessarily define a state of exhaustion in contrast to co-expression of multiple co-inhibitory receptors (38, 39). Interestingly, these co-expression patterns are functionally related, as a concurrent blocking of these multiple co-inhibitory receptors leads to synergistic reversal of exhaustion (40–42). For example, *in vitro* blocking of PD-1 alone failed to restore the functions of hepatic PD-1^+^CTLA-4^+^ HCV-specific CD8^+^ T-cells, but a concurrent blocking of CTLA-4 and PD-1 reinvigorated these cells in a CD4^+^ T-cell–independent manner (26).

The quality of T-cell response in the context of HCV viral autologous sequence in chronic infection is poorly understood, especially with respect to immune exhaustion. In particular, the relationship between PD-1/Tim-3 T-cell exhaustion phenotype, especially in the context of the co-expression of these receptors and the in-depth diversity of immune epitopes, is largely unknown. Furthermore, methodological limitations of conventional Sanger sequencing of cloned viral variants allow for the detection of major variants in a genetically diverse viral population. With the advent of next-generation sequencing (NGS), it is now possible to routinely detect variants present at low frequencies, which would remain undetected by standard sequencing methods (43, 44).

The diversity of viral epitopes and immune exhaustion represent major hindrances to spontaneous viral clearance and successful vaccine design (45). Since recent HCV vaccines failed in clinical trials, it is of major importance to elucidate the mechanisms behind successful HCV-specific immunity (46, 47). HCV infection represents a unique immunological experimental model, since it is one among few human viral infections with a dichotomous outcome (viral clearance *vs* chronic infection) and can be cured by highly specific small molecule drugs (45, 48). Thus, the aim of the present study was to determine whether T-cell exhaustion phenotype in chronic HCV infection is related to the sequence repertoire of viral immune epitopes. The analysis was restricted to immunodominant epitopes within NS3 viral gene: NS3_1073_ and NS3_1406_, which are commonly recognized in HLA-A^*^02-positive patients and NS3_1436_ which are recognized in HLA-A*01-positive patients. The study provides evidence that T-cell exhaustion phenotype in chronic HCV infection is related to the polymorphisms of HCV NS3 immune epitopes, but this is highly dependent on the restricting HLA allele context.

## Materials and Methods

### Patients

The study encompassed 90 prospectively enrolled patients with chronic HCV infection (anti-HCV^+^, HCV RNA^+^), presenting for treatment at the Outpatient Clinic of the Warsaw Hospital for Infectious Diseases. The source and timing of infection were unknown in most patients. However, all patients were HCV RNA positive for at least six months prior to therapy. All but one patient achieved sustained virologic response (SVR) (negative PCR test detecting HCV RNA six months post-treatment of sensitivity ≤15 IU/mL). Inclusion criteria were infection with genotype 1b, no evidence of cirrhosis, and no other potential cause of chronic liver disease. Thirty-six mL of EDTA-anti-coagulated whole blood was collected from all patients before treatment and six months post-treatment in the single non-responder. HCV genotype was determined by Inno-LiPA HCV II (Innogenetics N.V., Gent, Belgium) and baseline viral load as well as SVR status were assessed by RealTime HCV Viral Load Assay (Abbott) (sensitivity 12 IU/mL). Some clinical and virological characteristics of analyzed patients are presented in **Table 1**.

**Table 1:** Clinical, laboratory and virological characteristics of 90 patients with chronic hepatitis C.

Sex [male/female]	29/61
Age [years]	
median (range)	58.0 (25-88)
mean±SD	56.7±1.6
Serum ALT activity [U/mL]	
median (range)	61 (19-389)
mean±SD	79.1±6.3
normal values: 7–56 U/mL	
Fibrosis score determined by FibroScan^a^	F0/1, n=50
	F2, n=26
	F3, n=14
	F4, n=0
Viral load [U/mL]	
median (range)	8.4×10^5^ (6.2×10^3^ -1.1×10^7^)
mean±SD	1.4×10^6^±1.8×10^5^

a F0/F1 represents no or minimal fibrosis, F2 moderate fibrosis, F3 severe fibrosis, and F4 represents cirrhosis (49).

### Antibodies and Pentamers

Mouse anti-human anti-CD3-peridinin-chlorophyll protein-Cyanine5.5 (PERCP-CY5.5) clone UCHT1, anti-CD4-BD Horizon V500 clone RPA-T4, anti-PD-1-Alexa Fluor 647 clone EH12.1 and anti-Tim-3 (CD366)-Brilliant Violet (BV421) Clone 7D3, antibodies were purchased from BD Biosciences (Franklin Lakes, New Jersey, USA). Anti-CD8-fluorescein isothiocyanate (FITC) LT8 clone antibody and custom Pro5 Recombinant MHC class I Pentamer containing HLA-A*02-restricted HCV NS3_1406_ immunodominant epitope KLSGLGLNAV (corresponding to genotype 1b), conjugated with phycoerythrin (PE), were purchased from Pro-Immune (Oxford, United Kingdom). The latter epitope is one of the most immunogenic in chronically HCV-infected patients and displays cross-reactivity with other epitope variants (50, 51). BD Horizon Fixable Viability Stain 780 (BD Biosciences), an amine-reactive dye, was used to discriminate viable from non-viable lymphocytes. As isotype controls, IgG1 κ ALEXA 647 and IgG1 κ BV421 (BD Biosciences) were used.

### HLA-A Typing

The presence of the HLA-A*02 allele was verified by flow cytometry using anti-HLA-A*02-FITC clone BB7.2 antibody (BD Biosciences) and by quantitative PCR as described elsewhere (52). The presence of the HLA-A*01 allele was verified by flow cytometry using anti-HLA-A*01-biotin conjugated antibody and streptavidin-PE (both from United States Biological, Salem, USA) and by qualitative PCR as described previously (53).

### T-Cell Phenotyping

Peripheral blood mononuclear cells (PBMCs) were isolated from 36 ml of EDTA-anticoagulated blood by density gradient centrifugation using Lymphoprep, Stemcell Technologies Inc., Vancouver, British Columbia, Canada, according to the manufacturer’s protocol. After isolation, cells were passed through a 70 μm cell strainer (BD Biosciences), resuspended in Phosphate Buffered Saline pH 7.2 (Life Technologies, Carlsbad, USA), and counted. Next, 25 million freshly isolated PBMCs were stained with BD Horizon Fixable Viability Stain 780 (BD Biosciences), resuspended in the Pharmingen Stain Buffer with 0.2% (w/v) bovine serum albumin (BD Biosciences) and pre-incubated with FcR blocking reagent (Miltenyi Biotec, Bergisch Gladbach, Germany). Cells from HLA-A*02-positive patients were subjected to enrichment of HCV-specific CD8^+^ T-cells by magnetic separation. In brief, pentamer was added to the cells and the mixture was incubated for 10 minutes at room temperature in the dark. The cell suspension was then washed with 4 ml of MACS Buffer (Miltenyi Biotec). Cell pellets were resuspended with Anti-PE Micro Beads (Miltenyi Biotec) and MACS Buffer and incubated for 20 minutes at 4˚C, protected from light. The cell suspension was washed twice with MACS Buffer and passed through a 70 μm cell strainer (BD Biosciences). Magnetic MS Columns (Miltenyi Biotec) were used to perform the separation following the manufacturer’s recommendations. Enriched cells were counted and stained with anti-CD3, -CD4, -CD8, -PD-1, -Tim-3 antibodies for 20 minutes at 4°C. Controls consisted of unstained cells and fluorescence minus one (FMO) with Anti-IgG1 Alexa Fluor 647 and Anti-IgG1 BV421 instead of anti-PD-1 and anti-Tim-3, respectively. After washing twice with PBS, cells were resuspended in 300 μl of the Pharmingen Stain Buffer, immediately acquired on FACS Canto II instrument (Becton-Dickinson, Mountain View, USA) and analyzed by BD FACS Diva software (Becton-Dickinson). Typically, one million stained cells per sample were analyzed. Additionally, a separate PBMC sample (both from HLA-A*02-positive and HLA-A*02-negative subjects) was directly stained with antibodies against surface molecules (without pentamer staining step) and analyzed as above.

### Next-Generation Sequencing of Immune Epitopes

Diversity analysis of HLA-restricted NS3_1073_, NS3_1406_ and NS3_1436_ immunodominant epitopes contained within NS3/4a viral gene was conducted by next-generation amplicon sequencing. First, RNA was extracted from one mL of plasma using NucleoSpin RNA Virus-Kit (Macherey-Nagel, Düren, Germany), purified from any contaminating DNA using DNA-free DNA Removal Kit (Ambion, Austin, Texas, United States) and then subjected to reverse transcription using PrimeScript Reverse Transcriptase (Takara, Kusatsu, Shiga, Japan). Amplicon of 2223 bp encompassing the NS3/4a and containing NS3_1073_, NS3_1406_ and NS3_1436_ encoding regions (nt 3466-5689 of H77 reference genome, GenBank accession number AF009606) was obtained in two-step PCR using outer primers FW 5’-GGCGTGTGGGGACATCATC-3’ (nt 3314-3332), RV 5’-GGCTGTGAATGCCATCAGTGATG-3’ (nt 5704-5726) and inner primers FW 5’- GCATCATCACTAGCCTCACAGG-3’ (nt 3466-3487), RV 5’-CCAGGCAGAGTGGACAAGC-3’ (positions 5671-5689) and Platinum *Taq* DNA High Fidelity Polymerase on GeneAmp 9700 cycler (Applied Biosystems Foster City, California, USA). PCR conditions were as follows: initial denaturation at 94°C for 2 min, followed by 35 cycles of denaturation at 94°C for 30s, annealing at 55°C for 30s, and elongation at 68°C for 2 min 30s. Each PCR product was purified from agarose gel by Nucleospin Gel and PCR Clean-up Kit (Macherey-Nagel) and subjected to tagmentation and double indexing using Nextera XT Sample preparation Kit (Illumina, San Diego, California, United States). The run was performed on MiSeq (Illumina) platform using MiSeq Reagent v3 kit 2x300 bp, (Illumina).

### Data Analysis

For cytometric analyses, an initial lymphocyte gate was set based on side scatter (SSC)/forward scatter (FSC) and additional gates (single T-cells, live cells, CD3^+^, CD4^+^, CD8^+^, pentamer-positive, PD-1^+^, Tim-3^+^ cells) were introduced based on the appropriate controls of unstained cells and FMO.

For NGS analyses, amplicon sequence reads were filtered for quality (Phred score >20) yielding a depth of 53483.9 ± 18676 reads per sample covering epitopes NS3_1406_ and NS3_1436_ and 10119.4 ± 4758.2 per sample covering epitope NS3_1073_ (mean ± SD), corrected for sequencing errors and reconstructed into populations of genetic variants using Quasirecomb software (54). The latter uses a jumping hidden Markov model to infer *quasispecies* sequences along with their frequencies from the next-generation sequencing data. Based on the previously estimated sequencing error of the similar analysis (including MMLV-based reverse transcriptase, *Taq* polymerase, and Illumina sequencing), the maximal aggregate error rate would be about 1×10^−2^ per site (55) and thus, accordingly, one percent cutoff of frequency was applied to the reconstructed variants in order to exclude erroneous variants from the analysis. Next, the amino acid composition of epitopes variants (NS3_1073_, NS3_1406_ and NS3_1436_) was assessed using MEGA 6.0 software (56) and represented graphically using WebLogo generator (57). The GenBank EU255962.1 sequence was selected as the prototype 1b HCV strain, as it showed the highest similarity to patients’ epitope sequences. Variability of epitope was defined as the presence of at least two variants at a frequency >1%. Minor epitope variant was defined to be the second and subsequent most frequent in a sample and its frequency was between 1% and 30%.

### Statistical Analysis

Results were verified for normal distribution by the Kolmogorov–Smirnov test and expressed as mean values ± standard error (SE) or median (range). The Mann-Whitney U test/Fisher Exact Test was used to compare expression of immune exhaustion markers on CD8^+^ T-cells and viral epitope diversity parameters. Immune selection was assumed to be present when mutations within the viral epitope were more frequent in patients carrying the relevant HLA allele than in patients without the allele. All P-values were two-tailed and considered significant when ≤0.05.

### Ethical Statement

The study protocol followed ethical guidelines of the 2013 Declaration of Helsinki and was approved by the Bioethical Committee of the Medical University of Warsaw (Approval Number KB/77/A/2015). All patients provided written informed consent.

## Results

### Frequency of HLA-A*01 and HLA-A*02 Alleles

Forty (44.4%) patients displayed the presence of the HLA-A*02 allele, and 30 (33.3%) displayed the HLA-A*01 allele. Of these, five patients (5.6%) manifested the presence of both HLA-A*01 and HLA-A*02 alleles. Twenty-five patients (27.8%) were both HLA-A*01 and HLA-A*02-negative. The diversity analysis of HLA-A*02-restricted NS3_1073_, NS3_1406_ HCV epitopes and HLA-A*01-restricted NS3_1436_ HCV epitope was successful in all 90 patients.

### HLA-A*02-Restricted NS3_1073_ Epitope Was Moderately Conserved

NS3_1073_ epitope was moderately conserved, both at interhost (i.e., between patients) and intrahost (i.e., within patient) levels. Only in eight patients (8.9%) a maximum of two variants were present (major and minor variant), while in the remaining 82 (91.1%) a single variant was detected. The prototype NS3_1073_ CVNGVCWTV variant, representing the most common variant found of HCV 1b (e.g., GenBank EU255962.1) was present in 57 (63.3%) patients (in 55 as a single variant and in two as a minor variant). The remaining patients displayed the presence of various epitope sequences, among which: CINGVCWTV [previously described as a 1b escape variant (58)] was present as a major variant in 25 patients and as a minor variant in five; CINGVCWSV was a major variant in two patients; CINGACWTV was a major variant in three patients; CVNGACWTV was a major variant in two patients; CINGVCWTA, CVNGVCWSV, and CLNGVCWTV were major variants in one patient each, respectively; and CVNGVC*TV was a minor variant in one. Distribution of dominant aminoacid sequences in epitope NS3_1073_ among HLA-A*02-positive and HLA-A*02-negative patients are presented on **Supplementary Figure S1**.

**Figure 1:**
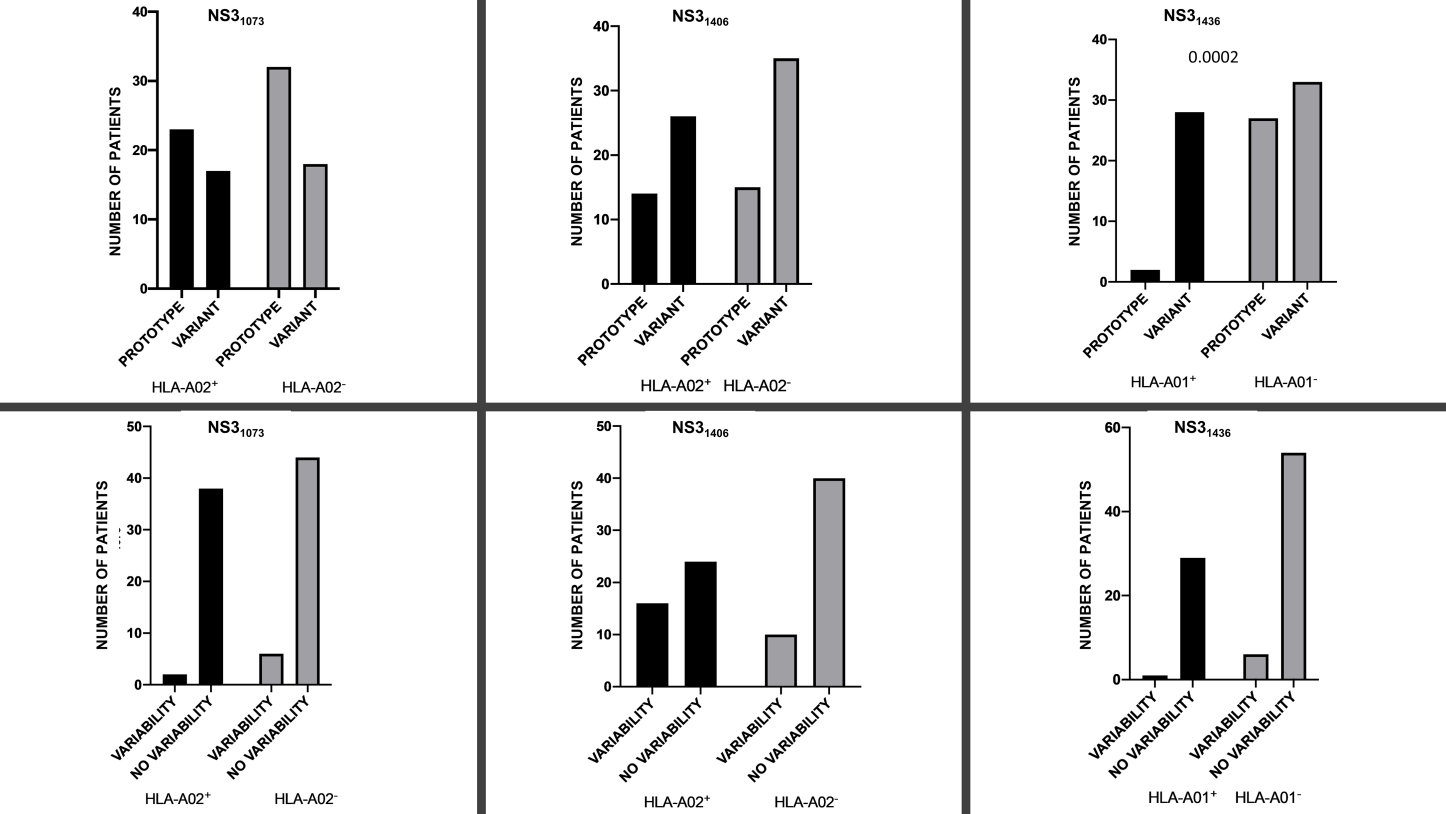
Prevalence of prototype 1b (GenBank EU255962.1) variant (upper panel) dominant sequence and presence/absence of variability (*i.e.*, ≥2 variants) (lower panel) for NS3_1073_, NS3_1406_ and NS3_1436_ epitopes in the context of the restricting HLA-A*01/HLA-A*02 alleles.

There was no difference in the presence of intrahost aminoacid variability at NS3_1073_ epitope in neither HLA-A*02-positive nor HLA-A*02-negative patients (2/38 *vs* 6/44, P=0.2920). Similarly, there was no significant difference between HLA-A*02-positive and HLA-A*02-negative patients in the prevalence of dominant prototype NS3_1073_ CVNGVCWTV *vs* variant epitope sequence (*i.e.*, other than prototype NS3_1073_ CVNGVCWTV as a dominant sequence) (n=23/17 *vs* 32/18), P=0.6638 (**Figure 1**).

### Infection With a Variant NS3_1073_ Epitope Was Associated With Lower PD-1/Tim-3 Expression on CD8^+^ T-Cells

**Figure 2:**
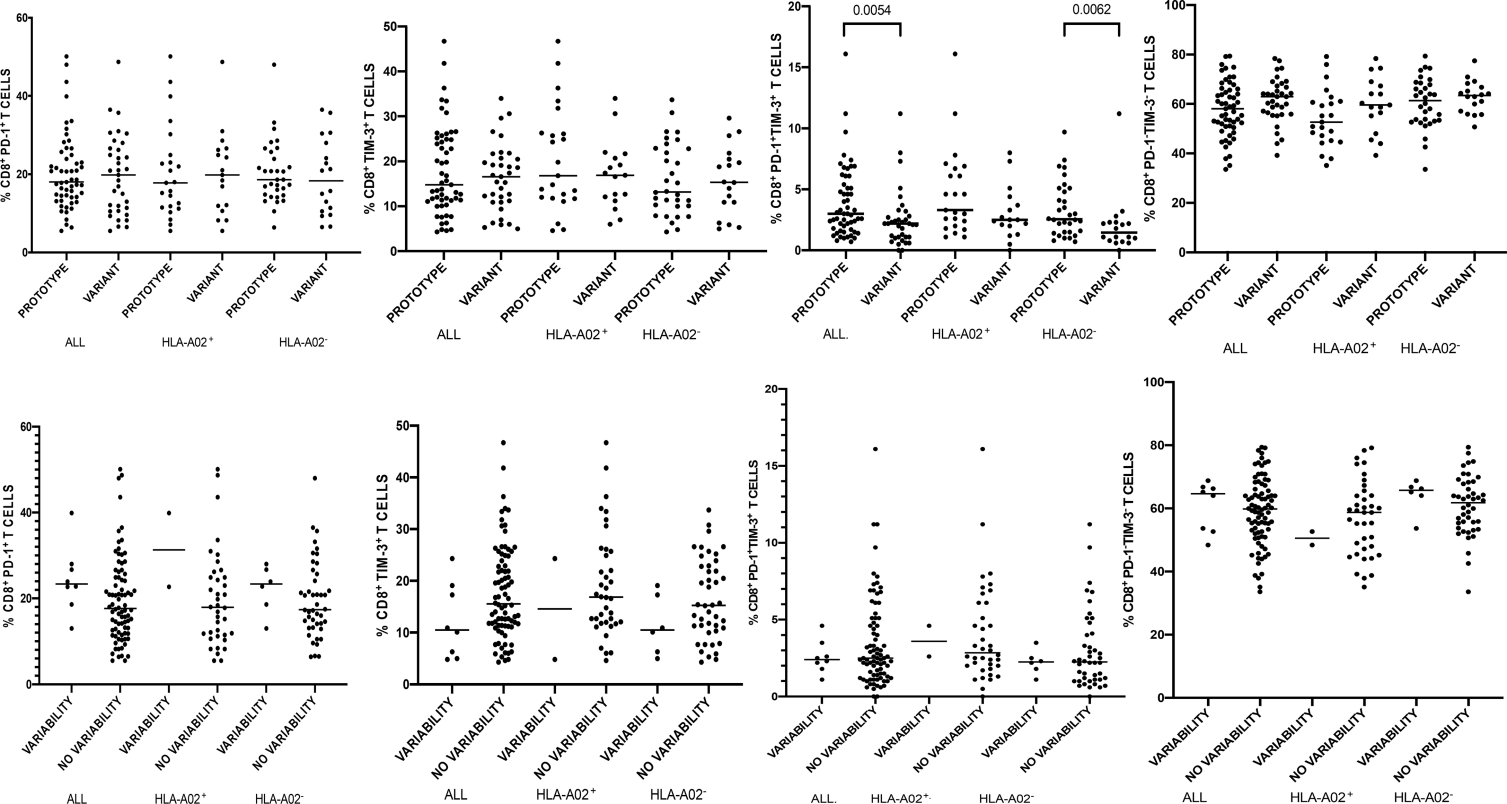
Percentages of peripheral blood CD8^+^ T-cells expressing PD-1/Tim-3 in patients infected with either NS3_1073_ prototype (GenBank EU255962.1) or variant epitope sequence as the dominant strain (upper panel). Lower panel shows intrahost aminoacid variability of this epitope where no variability denotes the presence of a single variant and variability indicates ≥ 2 variant sequences. Horizontal lines represent median values. Numbers above each bracket express P values.

When analyzing the entire cohort (*i.e.*, both HLA-A*02-positive and HLA-A*02-negative patients) there were no differences in the percentages of global CD8^+^ T-cells expressing either PD-1 and/or Tim-3 or negative for both PD-1 and Tim-3 in patients with presence/absence of intrahost aminoacid variability in the NS3_1073_ epitope. Similarly, no differences were present in either HLA-A*02-positive or HLA-A*02-negative subgroups (**Figure 2**).

When analyzing the entire cohort (i.e., both HLA-A*02-positive and HLA-A*02-negative patients) there were no differences in the percentages of global CD8^+^ T-cells expressing either PD-1 or Tim-3 or expressing neither PD-1 nor Tim-3 in patients harboring prototype or variant epitope as the dominant strain (**Figure 2**). However, infections with a variant epitope (i.e., other than the prototype NS3_1073_ CVNGVCWTV) as the major sequence were associated with significantly lower percentages of global CD8^+^ T-cells co-expressing PD-1 and Tim-3 (median 2.2 *vs* 3.0, P= 0.0054 (**Figure 2**). This was also true for both HLA-A*02-positive and HLA-A*02-negative subgroups, but the difference reached statistical significance only in the latter (1.45 *vs* 2.55, P=0.0062).

### HLA-A*02-Restricted NS3_1406_ Epitope Displayed High Level of Variability

NS3_1406_ epitope was the most variable, both at interhost and intrahost levels. The most prevalent NS3_1406_ epitope sequence [which was present in 35 (28.23%) of patients] was KLSGLGLNAV, consistent with the prototype 1b sequence (GenBank EU255962.1) and pentamer, followed by KLSSLGLNAV [previously shown to be cross-reactive with prototype sequence (50)] (n=27, 21.77%), KLSSLGINAV (n=13, 10.48%), KLSGLGINAV [cross-reactive with prototype (50)], (n=12, 9.68%), KLSALGINAV (n=5, 4.03%), KLSALGLNAV [cross-reactive with prototype (51)] and KLSSLGVNAV in three patients each (2.42%), KLLSLGINAV, KLSGLGLNAI, and QLSSLGLNAV in two patients each (1.61%), RLLALGINAV, QLSGLGVNAV, KLMGLGVNAV, KLSGLGMNAV, KLSSLSINAV, KLTALGINAV, KLSNLGINAI, KLLSLGLNAV, KLSTLGINAV, KLLALGINAV, KLLGLGINAV, KLVGLGVNAV, KLTALGLNAV, KLSGLGFNAV, KLSSLGVSAV, KLSSLGISAV, QSSSLGLNAV, KLSTLGLNAV, QLSSLGINAV, and KLSSLSIN-A in one patient each (0.8%). Distribution of dominant NS3_1406_ amino acid sequences in HLA-A*02-positive and HLA-A*02-negative patients is presented in **Supplementary Figure S2**.

Sixty-four (71.11%) patients displayed no variability within this epitope. Among the remaining 26 patients, two variants were present in 22 (24.44%), three in two (2.22%) and five variant sequences were present in the other two patients (2.22%). Only four amino acid positions (1407, 1410, 1413, 1414) were conserved among the analyzed variants (**Supplementary Figure S2**). The number of circulating variants was higher in HLA-A*02-positive than in HLA-A*02-negative patients (mean 1.6 *vs* 1.2), P=0.0210.

Prototype 1b NS3_1406_ KLSGLGLNAV was the dominant sequence in 14 out of 40 (37.5%) HLA-A*02-positive and in 15 out of 50 (30%) HLA-A*02-negative patients (P=0.6548; **Figure 1**). Similarly, there was no significant difference in the prevalence of domination of 1b prototype NS3_1406_ KLSGLGLNAV or cross-reactive KLSSLGLNAV, KLSGLGINAV or KLSALGLNAV sequence between HLA-A*02-positive (25 out of 40, 62.5%) and HLA-A*02-negative (35 out of 50, 70%) patients, P=0.5044. While HLA-A*02-positive patients were more likely to display intrahost aminoacid variability in the NS3_1406_ epitope (≥ 2 sequences), this difference did not reach statistical significance (P=0.0601; **Figure 1**).

### NS3_1406_ Epitope Sequence Variability Was Associated With Higher PD-1/Tim-3 Expression on CD8^+^ T-Cells

**Figure 3:**
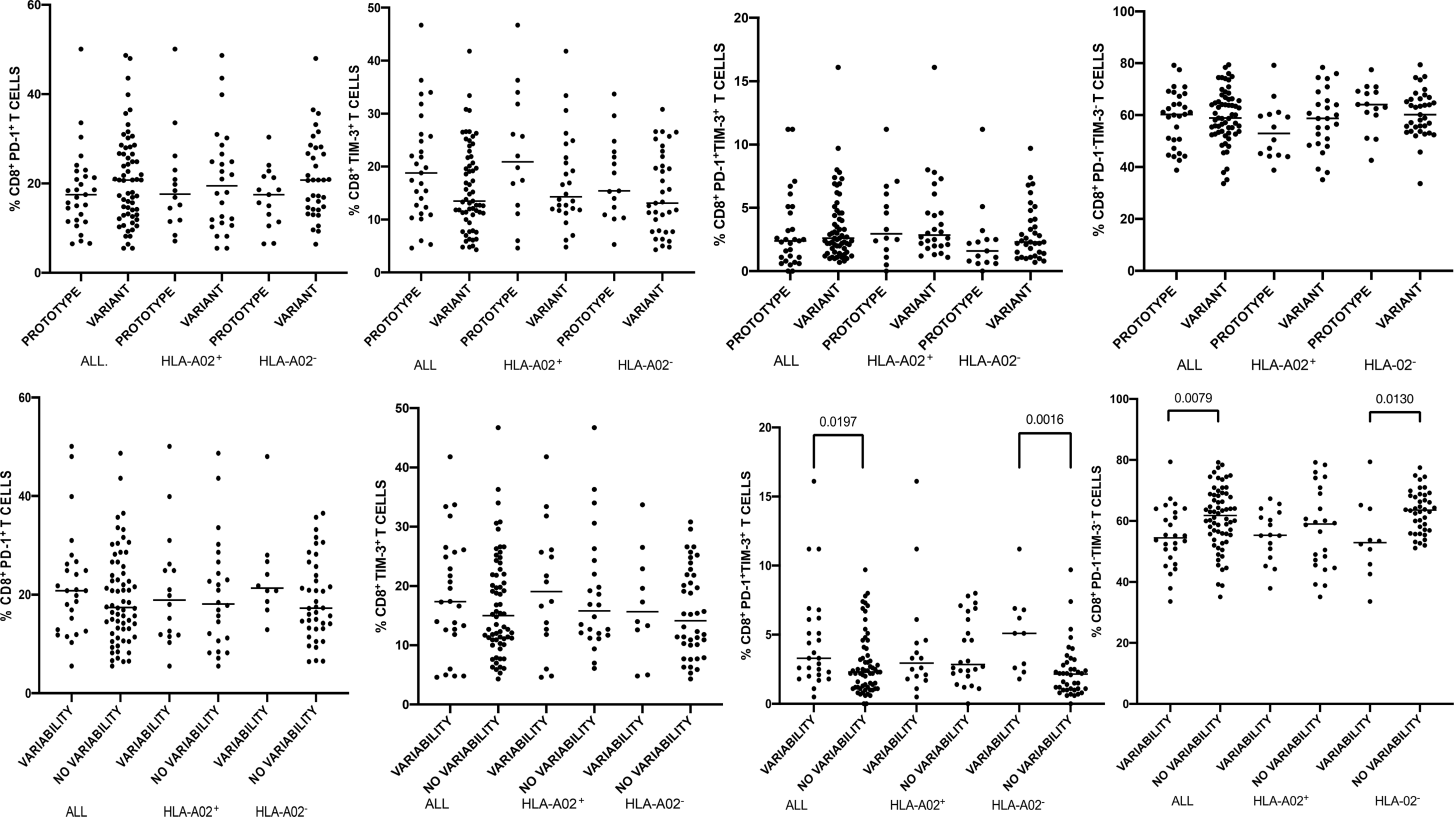
Percentages of peripheral blood CD8^+^ T-cells expressing PD-1/Tim-3 in patients infected with either NS3_1406_ prototype (GenBank EU255962.1) or variant epitope sequence as the dominant strain (upper panel). Lower panel shows intrahost aminoacid variability of this epitope where no variability denotes the presence of a single variant and variability indicates ≥ 2 variant sequences. Horizontal lines represent median values. Numbers above each bracket express P values.

Variability of NS3_1406_ epitope was not associated with PD-1, Tim-3 and PD-1 and Tim-3 expression on global peripheral CD8^+^ T-cells in HLA-A*02-positive subjects, but when all enrolled patients were analyzed, variability (≥ 2 variants) was associated with increased percentage of global CD8^+^ PD-1^+^Tim-3^+^ T-cells (P=0.0197) and with lower percentage of CD8^+^ T-cells negative for both exhaustion markers (P=0.0079) (**Figure 3**). A similar relationship was found in HLA-A*02-negative subjects: NS3_1406_ epitope variability was associated with higher percentage of global CD8^+^ PD-1^+^Tim-3^+^ T-cells (P=0.0016) and lower percentage of CD8^+^ PD-1^−^Tim-3^−^ T-cells (P=0.0130) (**Figure 3**).

When analyzing the entire cohort and either HLA-A*02-positive or HLA-A*02-negative patients, there were no statistically significant differences in the percentages of global CD8^+^ T-cells expressing either PD-1 and/or Tim-3 or neither PD-1 nor Tim-3 between patients with the prototype NS3_1406_ KLSGLGLNAV or variant epitope as a dominant sequence (**Figure 3**).

### NS3_1406_ Epitope Sequence Other Than Prototype or Cross-Reactive Variants Was Associated With Higher PD-1/Tim-3 Expression on HCV-Specific CD8^+^ T-Cells

**Figure 4:**
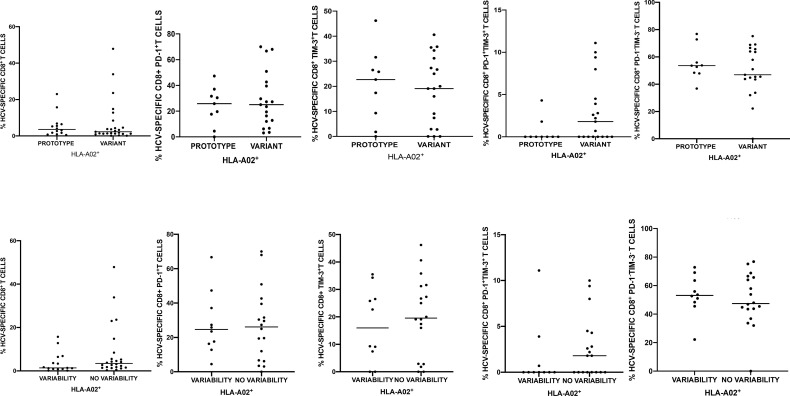
Percentages of peripheral blood HCV-specific CD8^+^ T-cells expressing PD-1 and Tim-3 in 40 HLA-A*02-positive patients harboring NS3_1406_ prototype (GenBank EU255962.1) or variant epitope sequence as the dominant strain (upper panel). Lower panel shows intrahost aminoacid variability of this epitope where no variability denotes the presence of a single variant and variability indicates ≥ 2 variant sequences. Horizontal lines represent median values.

In HLA-A*02-positive subjects there was no association between the variability within the NS3_1406_ epitope and the percentage of HCV-specific cells or PD-1/Tim-3 expression on these cells. Furthermore, patients in whom the prototype sequence dominated were not different with respect to the above parameters from the other patients (**Figure 4**). However, predominance of an epitope sequence representing neither prototype NS3_1406_ KLSGLGLNAV nor cross-reactive variants (KLSSLGLNAV, KLSGLGINAV or KLSALGLNAV) was associated with significantly higher percentage of HCV-specific CD8^+^ T-cells with co-expression of PD-1 and Tim-3, P= 0.0102.

### HLA-A*01 -Restricted NS3_1436_ Epitope Was Highly Conserved

NS3_1436_ epitope was the most conserved both at interhost and intrahost levels as only two sequence variants were identified (ATDALMTGY and ATDALMTGF) and only in 7 patients (7.8%) ≥ 2 sequences were present. The prototype ATDALMTGY NS3_1436_ variant (GenBank EU255962.1), representing the most common variant found in HCV 1b, was present in 34 (37.8%) patients, (in 29 as a single variant and in five as a minor variant). ATDALMTGF, which was previously described in both prospective studies of primary infection and in cross-sectional studies of chronic disease as a viral escape variant in HLA*A1-positive patients (6, 59), was present in 61 patients as a sole variant and in two as a minor variant (70.0% of patients overall).

There was no difference in the presence of intrahost aminoacid variability among either HLA-A*01-positive or HLA-A*01-negative patients (one out of 30 *vs* 6 out of 60, respectively P= 0.4170), **Figure 1**. However, in almost all HLA-A*01-positive patients (28 out of 30, 93.3%), the ATDALMTGF escape sequence predominated, while in patients without this allele this was much less common (33 out of 60, 55%, P= 0.0002 (**Figure 1**). Interestingly, in HLA-A*01-positive patients all minor variants were prototypes while in HLA-A*01-negative patients, two were escape variants and one was a prototype. Distribution of dominant NS3_1436_ sequence in HLA-A*01-positive and in HLA-A*01-negative patients is presented on **Supplementary Figure S3**.

### NS3_1436_ Epitope Sequence Variation and CD8^+^ T-Cell PD-1/Tim-3 Expression Phenotype

When analyzing the entire cohort (i.e., both HLA-A*01-positive and HLA-A*01-negative patients), there were no significant differences in the percentages of global CD8^+^ T-cells expressing either PD-1 and/or Tim-3 or neither PD-1 nor Tim-3 in patients with the presence and absence of intrahost aminoacid variability within NS3_1436_ epitope (**Supplementary Figure S4**). Similarly, no significant differences were present in the HLA-A*01-negative subgroup, while in HLA-A*01-positive patients there were too few values for a meaningful statistical comparison.

When all patients harboring either prototype ATDALMTGY or variant epitope ATDALMTGF as the dominant sequence were compared, there were no differences in the percentages of global CD8^+^ T-cells expressing either PD-1 and/or Tim-3 or expressing neither PD-1 nor Tim-3 (**Supplementary Figure S4**). This comparison could not be done for the HLA-A*01-positive subgroup, as only two patients were infected with ATDALMTGY. However, these two patients displayed lower PD-1 and higher Tim-3, as well as PD-1 + Tim-3 expression, on global CD8^+^ T-cells than patients harboring HCV ATDALMTGF variant (**Supplementary Figure S4**).

Within HLA-A*01-negative patients, there were no differences in the expression of exhaustion markers on global CD8^+^ T-cells between patients infected with different variants (**Supplementary Figure S4**).

### Evolution of Epitopes and Exhaustion Markers in the Patient Not Responding to Treatment

We investigated dynamics of exhaustion markers in the context of viral epitopes variability in the only treatment-naïve non-responder patient treated for 8 weeks with Harvoni. Despite negative viral load at week 4 and 8 of treatment, this patient experienced viral load rebound between the end of treatment and SVR assessment six months post-treatment. His viral load was 821000 before and 643000 IU/ml after treatment. The patient was HLA-A*02-positive, HLA-A*01-negative. Initially (*i.e*., prior to treatment), this patient displayed the presence of NS3_1073_ escape epitope CINGACWTV which reverted to prototype CVNGVCWTV after unsuccessful treatment. Concomitantly, there was a change in NS3_1406_ sequence from KLSALGLNAV to QLSSLGLNAV. Furthermore, despite the absence of HLA-A*01 allele in this patient, the viral escape NS3_1436_ epitope sequence ATDALMTGF reverted to the prototype ATDALMTGY (**Supplementary Figure S5**). All these post-treatment variants were not detected prior to treatment even below the cutoff value of 1%. Concomitantly, the frequency of global CD8^+^ T-cells expressing either PD-1 and/or Tim-3 have decreased and the frequency of CD8^+^ global T-cells expressing neither PD-1 nor Tim-3 increased (**SupplementaryFigure S5**).

## Discussion

The aim of the present study was to determine whether T-cell exhaustion phenotype in chronic HCV infection is related to the repertoire of viral immune epitopes. According to our best knowledge, this is the first study of its kind. We studied a large prospective cohort of chronic hepatitis C patients infected with the same viral subgenotype (1b), as it was previously shown that the patterns of viral adaptation to host’s immune pressure reflected by HLA-associated viral epitope polymorphisms may vary considerably between the genotypes, despite the presence of the same HLA allele (60). We assessed exhaustion phenotype at a single-cell level on both global CD8^+^ and HCV-specific T-cells employing multiparametric flow cytometry combined with magnetic-based enrichment of the latter cells using MHC-pentamers.

We found that the PD-1/Tim-3 inhibitory receptors expression pattern is associated with specific autologous viral epitope sequence and its intrahost variability and this was evident for both HCV-specific and global CD8^+^ T-cell populations. Importantly, the type of association seemed to be epitope-specific, which warrants evaluation of autologous viral epitope sequences when investigating CD8^+^ T-cell exhaustion.

Although viral pathogens encode numerous potentially immunogenic determinants, CD8^+^ T-cells recognize and respond only to a very small fraction of these potential epitopes. This phenomenon is called immunodominance and is strongly restricted by particular HLA alleles (61). Immunodominant HCV-specific CD8^+^ T-cell epitopes located in E2, NS3 or NS5B are targeted in the vast majority of patients expressing the respective HLA type (62–64). Available studies of natural HCV infection and using adenovirus-based vaccine immunogens revealed that NS3 immunodominant CD8^+^ T-cell responses to HCV 1b include NS3_1406_ KLSGLGINAV, NS3_1073_ CVNGVCWTV restricted by HLA class-I A*02 allele, and NS3_1436_ ATDALMTGY restricted by HLA-A*01 alleles (6, 16, 17, 50, 59, 65). Since these two HLA-A alleles are the most prevalent in the Polish population (44.4% of patients were HLA-A*02-positive, 33.3% patients were HLA-A*01-positive), we narrowed the analysis to immunodominant epitopes NS3_1073_, NS3_1406_ and NS3_1436_.

The analyzed epitopes displayed variable levels of amino acid diversity. The most variable both at interhost and intrahost levels was NS3_1406_ epitope, suggesting the highest tolerance of this region to amino acid substitutions (**Supplementary Figure S2**). This is consistent with the study by Kelly et al. in which epitope NS3_1406_ was highly variable, with single amino acid sequence present in only 25% of genotype 1 infected individuals (50). In contrast, in our study epitope NS3_1073_ was only moderately variable (**Supplementary Figure S1**) and NS3_1436_ displayed almost no variability (**Supplementary Figure S3**), which is consistent with previous observations (22) and implies strong functional constraints and high “fitness cost” related to viral regions encoding these epitopes. Viral escape mutations of high ‘‘fitness cost’’ have been previously observed in some epitopes with a limited sequence variability in the NS3/NS4a region (66). Mutations at positions 1074, 1075, 1076 and 1079 of the NS3_1073_ epitope were reported to negatively affect the replicative fitness of the virus (66) and thus some escape mutations within CD8^+^ T-cell epitopes may be restricted due to incompatibility with replicative viral capacity (67–69). Consequently, certain viral escape mutations can only evolve together with compensatory mutations that retain viral replication (70, 71).

Importantly, our study showed that immunodominance exerts selective pressure within the analyzed NS3 epitopes, since there were statistically significant differences in epitope sequence variability and/or their number (complexity) in the presence or absence of the restricting HLA-A allele. In particular, in patients harboring HLA-A*02 allele, the number of NS3_1406_ epitope variants was higher than in HLA-A*02-negative patients. Similarly, the HLA-A*01 allele was associated with escape of the NS3_1436_ epitope, since almost all HLA-A*01-positive patients displayed dominant ATDALMTGF escape epitope sequence (Y1444F substitution), while patients without this allele displayed either dominant escape ATDALMTGF or prototype ATDALMTGY (**Supplementary Figure S3**). This suggests that HLA-A*01-positive subjects quite commonly target this epitope. In line with this hypothesis, it was previously found that 39% of HLA-A*01-positive subjects had a detectable *ex vivo* response against this epitope (59). It was reported that the Y1444F substitution impairs binding to the HLA-A*01 molecule and is sufficient to abrogate CD8^+^ T-cell recognition, which may have an important impact on the ability to prime a functional response upon infection (72). On the other hand, this mutation was shown to have a negative impact on viral fitness, since a helicase activity of the protein containing the Y1444F substitution is reduced when compared to the prototype sequence (72). Interestingly, our study showed that minor epitope variants in HLA-A*01-positive patients were exclusively prototypes while in HLA-A*01-negative patients these were mostly escape variants which, again, may indicate immune-driven selection of this epitope and high fitness cost of the Y1444F substitution.

Previously, the Y1444F substitution has been observed in association with the HLA-A*01 allele in subjects with chronic infection indicating that mutational escape is common (16, 59). This may confirm the presence of the previously reported HLA class I associated epitope sequence polymorphisms, so called “HLA class I footprints”, observed on a population level and manifesting as mutations in autologous viral sequences only in patients positive for the restricting HLA class I allele (10, 14, 16, 59, 60, 73, 74). Moreover, it was revealed that, at least for some CD8 epitopes, identical escape mutations are selected in subjects sharing the same restricting HLA class I alleles (16, 59, 74, 75). The first evidence for selective pressure by CD8^+^ T-cells comes from studies on chimpanzees sharing the same MHC class I alleles and persistently infected with HCV. These studies demonstrated that mutations within viral epitopes occur shortly after infection and are not random, as the rate of nonsynonymous substitutions was higher in MHC class I restricted epitopes compared to non-restricted epitopes or nearby regions (76). For example, within NS3_1073_, NS3_1131_ and NS3_1169_ epitopes, the mutation rate was 10-fold higher than in conserved regions (77). Similarly, analysis of an Irish outbreak cohort has shown that mutations within the known HLA class I-restricted epitopes were more common than mutations at other sites (74). Furthermore, mutations in these epitopes were more frequent in the presence of particular alleles, pointing to immune selection (74). Studies using a population-based approach have shown that most viral escape mutations revert to wild-type prototype sequence upon transmission to a new host negative for the restricting HLA class I allele because of the high fitness cost incurred by these mutations (15, 78). The fact that these mutations are stable in chronic HCV- infected subjects suggests that selective pressures still operate during the chronic phase of infection (15).

Our study suggests that there was an interplay between the analyzed epitope sequences and the exhaustion phenotype of CD8^+^ T-cells, reflected by PD-1/Tim-3 expression. In particular, in HLA-A*02-positive patients the predominance of an epitope sequence representing neither prototype NS3_1406_ KLSGLGLNAV nor cross-reactive variant (KLSSLGLNAV, KLSGLGINAV or KLSALGLNAV) was associated with significantly higher percentage of HCV-specific cells CD8^+^ T-cells with co-expression of PD-1 and Tim-3. Given that it is the co-expression of PD-1 and Tim-3 that defines the state of more profound exhaustion (40, 41), these results indicate that variations from the genotype-specific consensus sequence may be associated with immune exhaustion.

When analyzing the entire cohort, i.e., both HLA-A*02-positive and HLA-A*02-negative patients, NS3_1406_ intrahost variability (i.e., the presence of at least two epitope variants) was associated with increased frequencies of global CD8^+^ T-cells with PD-1 and Tim-3 co-expression, and with lower frequencies of CD8^+^ T-cells without these exhaustion markers (**Figure 3**). This suggests that the higher exhaustion is related to the presence of multiple variants, possibly due to limited immune surveillance and natural viral evolution. However, this observation was not confirmed in the subgroup of HLA-A*02-positive patients in whom this epitope should be recognized, as the analyzed group was probably too small.

In the case of NS3_1073_ epitope, infection with a variant epitope (i.e., other than the genotype-specific prototype) was associated with significantly lower percentage of global CD8^+^ T-cells with co-expression of PD-1 and Tim-3, implying lower exhaustion (**Figure 2**). While this was statistically significant for the entire group (both HLA-A*02-positive and HLA-A*02-negative patients), it was not for the subgroup of HLA-A*02-positive patients in whom this epitope should be recognized, possibly due to too small number of patients analyzed. A similar phenomenon was observed in the case of NS3_1436_ epitope where HLA-A*01-positive patients harboring NS3_1436_ escape sequence displayed higher percentages of PD-1^+^ CD8^+^ T-cells and lower percentages of Tim-3^+^ as well as PD-1^+^Tim-3^+^ CD8^+^ T-cells than patients with the prototype sequence (**Supplementary Figure S4**). However, these data should be interpreted with caution due to the limited number of observations.

Although there are only a few studies investigating PD-1/Tim-3 expression on CD8^+^ T-cells in the context of epitope variability in HCV infection, some studies showed that the phenotype of HCV-specific CD8^+^ T-cells is determined by the level of antigen-specific stimulation. Expression of memory marker CD127 defines CD8^+^ T-cells that do not recognize cognate antigen because of viral variation (79). Similarly, Bengsch et al. found that co-expression of CD8^+^ inhibitory receptors such as 2B4, CD160 and KLRG1 in association with PD-1 represented exhausted phenotype and was associated with low and intermediate levels of CD127 expression, an impaired proliferative capacity, an intermediate T-cell differentiation stage, and an absence of sequence variations within the corresponding viral epitopes, indicating ongoing antigen triggering (38). Noteworthy, opposite expression profiles of inhibitory receptors were observed within the same patient depending on the autologous epitope sequence: in the presence of NS3_1406_ epitope escape all NS3_1406_ specific CD8^+^ T-cells expressed CD127 and low levels of PD-1, 2B4, CD160 and KLRG1, whereas an opposite phenotype was observed in case of NS5_2594_ specific CD8^+^ T-cells, which were characterized by low CD127 expression, but high PD-1, 2B4, CD160 and KLRG1 (38). Similar studies on HIV-1 and SIV infection revealed high level of PD-1 expression upon recognition of the cognate epitope and subsequent decrease after *in vivo* selection of cytotoxic T lymphocyte escape mutations in the respective epitopes (80–82). These results again imply that the mechanisms responsible for inhibitory receptor expression operate in an epitope-specific manner depending on the autologous virus sequence. Thus, both viral escape and T-cell exhaustion, defined by the expression of multiple inhibitory receptors, seem to contribute to the ineffective viral control present in chronic HCV infection. Currently, it is unclear whether HCV epitopes sequence variation drives the T-cell exhaustion observed in chronic HCV infection or whether it is the consequence of exhausted T-cell phenotype and ensuing loss of viral control.

Administration of anti-HCV treatment may change the repertoire of the viral *quasispecies* because of the new selective drug-related pressure acting on the virus (83). Consequently, unsuccessful therapy may be related to alteration of the composition of the viral variants, which would affect the T-cell exhaustion phenotype. Therefore, by studying the evolution of the viral antigens driven by treatment, we can verify how this affects T-cell exhaustion. Thus, we aimed to investigate the dynamics of exhaustion markers expression in the context of viral epitopes sequence in one treatment-naïve HLA-A*02-positive, HLA-A*01-negative patient who experienced a viral relapse between the end of 8-week treatment with Harvoni and SVR assessment, and thus represented a unique case because of otherwise highly effective treatment. Based on epidemiological data, this patient was unlikely to be re-infected with a different HCV strain. Interestingly, reversal of escape NS3_1073_ epitope CVNGVCWTV to prototype CVNGVCWTV after treatment was accompanied by change of NS3_1406_ sequence from KLSALGLNAV to QLSSLGLNAV (**Supplementary Figure S5**). Furthermore, despite the absence of HLA-A*01 allele, NS3_1436_ escape epitope sequence ATDALMTGF changed to the prototype ATDALMTGY. Importantly, these post-treatment variants were not detected prior to treatment at any frequency, which suggests the presence of pre-existent minor strain at a very low frequency, or even *de novo* evolution. Concomitantly, the exhaustion markers expression on global CD8^+^ T-cells has decreased. Although multiple other factors could have contributed to the decrease of exhaustion phenotype after treatment, these results may also indicate that epitope sequence is a factor affecting exhaustion phenotype. In this case, loss of the escape variant (possibly due to treatment-related pressure) and appearance of prototype sequence (possibly a more replication-competent form) could have contributed to the reinvigoration of T-cell response after treatment. This could be because CD8^+^ T-cells targeting escaped epitopes were not exposed to constant T-cell receptor stimulation anymore and thus acquired a memory-like state rather than an exhaustion phenotype and sustained proliferative potential (38, 79). Similarly, in the study of Wieland et al., viral relapse and thus antigen re-exposure led to phenotypic and qualitative changes including a vigorous expansion of HCV NS3_1073_-specific CD8^+^ T-cells that was accompanied by re-generation of terminally exhausted effector subsets (84). These results also suggest that CD8^+^ T-cells are able to re-expand efficiently in response to antigen re-exposure.

Despite being the largest of its kind, our study has several limitations. First of all, with the exception of one patient, the analysis was confined to a single time point and thus the changes of CD8^+^ T-cell exhaustion markers and HCV NS3 epitope variant sequences over time remain unknown. Furthermore, our study was focused on phenotypical rather than functional markers of CD8^+^ T-cell exhaustion and the functional status of these cells remains to be determined.

Next, the analysis was confined to patients infected with subgenotype 1b, which, while reducing the confounding effects of varied genotypes, implies that the findings are not necessarily valid for other genotypes due to epitope polymorphism (6, 58, 60, 79).

It is known that various immune cells may support low level HCV replication. While CD8^+^ T-cells are relatively rarely infected (85, 86) it cannot be ruled out that infection of these cells *per se* and/or the presence of unique, lymphotropic viral variants, could have affected their exhaustion status. Undoubtedly, elucidating the effects of extrahepatic HCV infection on CD8^+^ T-cells exhaustion status would offer an interesting future direction of research.

Finally, although all patients fulfilled the criteria for chronic infection, having been HCV RNA positive for at least 6 months prior to the study, the exact duration of infection was unknown for the majority of them. However, this is not unusual as symptomatic manifestation of *de novo* infection is rare, subclinical course of the disease is the norm, and potential exposure (*e.g.*, parenteral drug abuse, infected family member, infected sex partner) is often spread over many years. Furthermore, in a substantial proportion of patients the route of transmission remains unknown as no risk factors are identified (87). Transfusion of blood or blood products, once the major source of HCV infection, became extremely rare in the developed countries after the introduction of sensitive tests for blood screening in 1992 (88).

## Conclusions

In summary, our study showed that analyzed NS3 epitopes are exposed to HLA-A allele related selective pressure, which was manifested in the respective restricting epitope sequence polymorphisms (escape of the NS3_1436_ epitope in HLA-A*01-positive patients) or intrahost variability (higher number of NS3_1406_ epitope variants in HLA-A*02-positive patients). Furthermore, our results provide evidence that the PD-1/Tim-3 inhibitory receptors expression pattern is associated with specific autologous viral epitope sequence and/or its intrahost variability and is evident for both HCV-specific and global CD8^+^ T-cell populations. Importantly, the type of observed association seems to be epitope specific. In particular, infection with predominant NS3_1406_ epitope representing neither prototype nor cross-reactive sequence was associated with higher percentage of CD8^+^ PD-1^+^Tim-3^+^ HCV-specific T-cells. Our study points to the importance of evaluating autologous viral epitope sequences in the investigation of CD8^+^ T-cell exhaustion in HCV infection.

## Data Availability Statement

The NGS datasets presented in this study can be found in the NCBI SRA BioProject, accesion no: PRJNA795441.

## Ethics Statement

The study protocol followed ethical guidelines of the 2013 Declaration of Helsinki and was approved by the Bioethical Committee of the Medical University of Warsaw (Approval Number KB/77/A/2015). All patients provided a written informed consent.

## Author Contributions

Conceptualization: KC, MR, and TL. Data curation: SO, KP, and HB. Data analysis: KC, SO, KP, AP, and MZ. Funding acquisition: KC. Investigation: SO, KC, and IB-O. Methodology: SO, KC, MR, and HB. Software: KP, KC, and MZ. Supervision: MR, TL, and KC. Manuscript review: SO, TL, KP, HB, IB-O, AP, MZ, MR, and KC. All authors contributed to the article and approved the submitted version.

## Funding

This study was funded by grant UMO-2015/19/D/NZ6/01303 from the National Science Centre, Poland. SO PhD scholarship was covered from the European Social Fund program POWR.03.02.00-00-I041/16-00.

## Conflict of Interest

The authors declare that the research was conducted in the absence of any commercial or financial relationships that could be construed as a potential conflict of interest.

## Publisher’s Note

All claims expressed in this article are solely those of the authors and do not necessarily represent those of their affiliated organizations, or those of the publisher, the editors and the reviewers. Any product that may be evaluated in this article, or claim that may be made by its manufacturer, is not guaranteed or endorsed by the publisher.

## Supplementary Material

Supplementary Figure S1 Distribution of dominant aminoacid sequences in epitope NS3_1073_ among HLA-A*02-positive and HLA-A*02-negative patients. Amino acids are colored according to their chemical properties: polar amino acids **(G, S, T, Y, C, Q, N)** are green, basic **(K, R, H)** blue, acidic **(D, E)** red, and hydrophobic **(A, V, L, I, P, W, F, M)** amino acids are black. Height of letters within the stack indicates the relative frequency of each amino acid at this position. Sequence logos were generated using WebLogo generator (57).

Supplementary Figure S2 Distribution of dominant aminoacid sequences in epitope NS3_1406_ among HLA-A*02-positive and HLA-A*02-negative patients. Amino acids are colored according to their chemical properties: polar amino acids **(G, S, T, Y, C, Q, N)** are green, basic **(K, R, H)** blue, acidic **(D, E)** red and hydrophobic **(A, V, L, I, P, W, F, M)** amino acids are black. Height of letters within the stack indicates the relative frequency of each amino acid at this position. Sequence logos were generated using WebLogo generator (57).

Supplementary Figure S3 Distribution of dominant aminoacid sequences in epitope NS3_1436_ among HLA-A*01-positive and HLA-A*01-negative patients. Amino acids are colored according to their chemical properties: polar amino acids **(G, S, T, Y, C, Q, N)** are green, basic **(K, R, H)** blue, acidic **(D, E)** red and hydrophobic **(A, V, L, I, P, W, F, M)** amino acids are black. Height of letters within the stack indicates the relative frequency of each amino acid at this position. Sequence logos were generated using WebLogo generator (57).

Supplementary Figure S4 Percentages of peripheral blood CD8^+^ T-cells expressing PD-1/Tim-3 in 90 patients infected with either NS3_1436_ prototype (GenBank EU255962.1) or variant epitope sequence as the dominant strain (upper panel). Lower panel shows intrahost aminoacid variability of this epitope where no variability denotes the presence of a single variant and variability indicates ≥ 2 variant sequences. Horizontal lines represent median values.

Supplementary Figure S5 Evolution of dominant aminoacid sequences in epitope NS3_1073,_ NS3_1406_, NS3_1436_ and percentages of CD8^+^ T-cells expressing PD-1/Tim-3 in the non-responder to treatment. PRE-TX-before treatment, POST-TX- post-treatment. Amino acids are colored according to their chemical properties: polar amino acids **(G, S, T, Y, C, Q, N)** are green, basic **(K, R, H)** blue, acidic **(D, E)** red and hydrophobic **(A, V, L, I, P, W, F, M)** amino acids are black. Sequence logos were generated using WebLogo generator (57).
