# Supplementary figures and images for: CD8+ T-Cell Exhaustion Phenotype in Chronic Hepatitis C Virus Infection Is Associated With Epitope Sequence Variation
Source: Front Immunol. 2022 Mar 21;13:832206. doi: 10.3389/fimmu.2022.832206 (PMC8977521; doi:10.3389/fimmu.2022.832206)

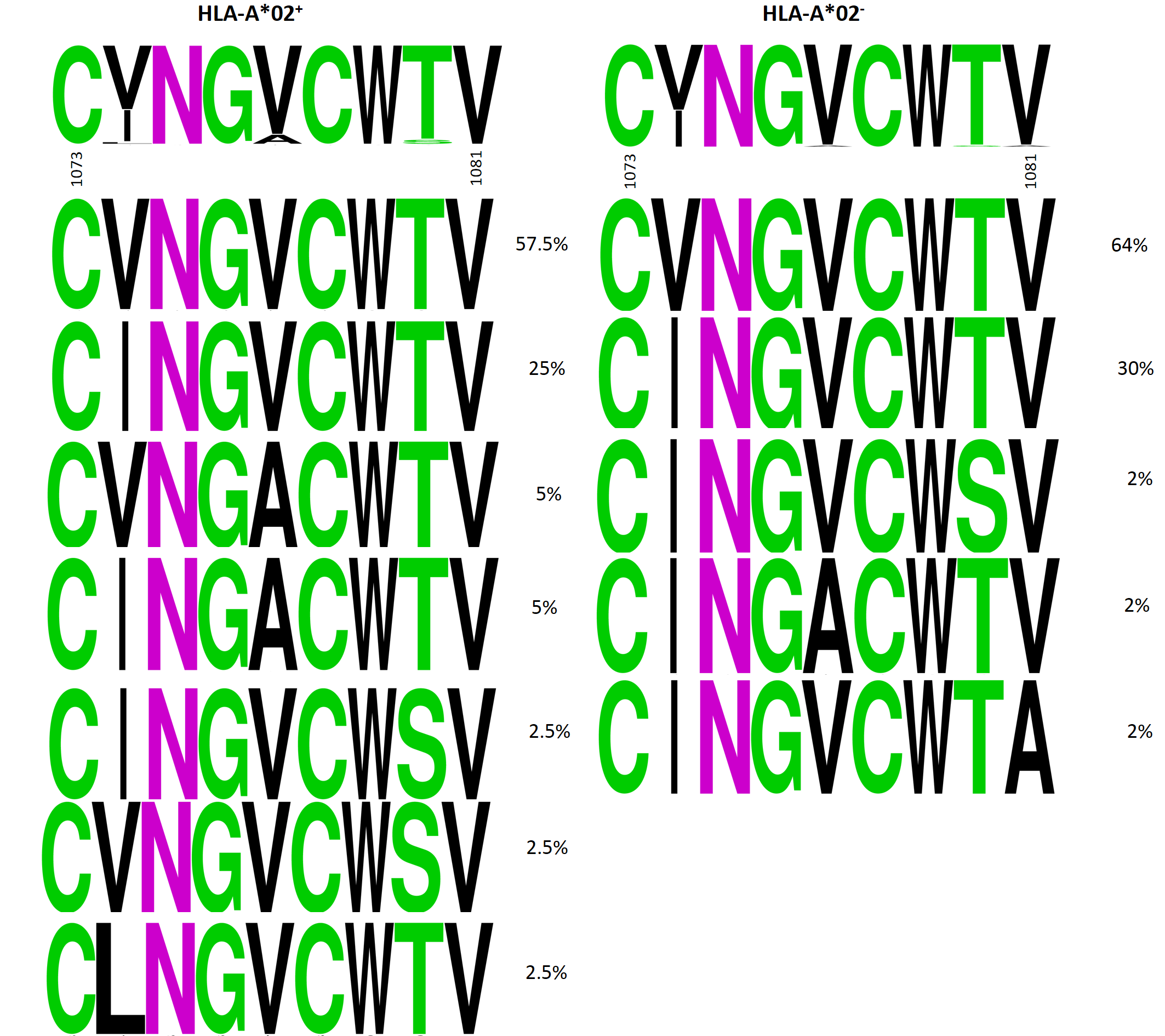

Supplement: Supplementary Figure S1 — Distribution of dominant aminoacid sequences in epitope NS31073 among HLA-A*02-positive and HLA-A*02-negative patients. Amino acids are colored according to their chemical properties: polar amino acids (G, S, T, Y, C, Q, N) are green, basic (K, R, H) blue, acidic (D, E) red, and hydrophobic (A, V, L, I, P, W, F, M) amino acids are black. Height of letters within the stack indicates the relative frequency of each amino acid at this position. Sequence logos were generated using WebLogo generator (57). [file Image_1.tif]

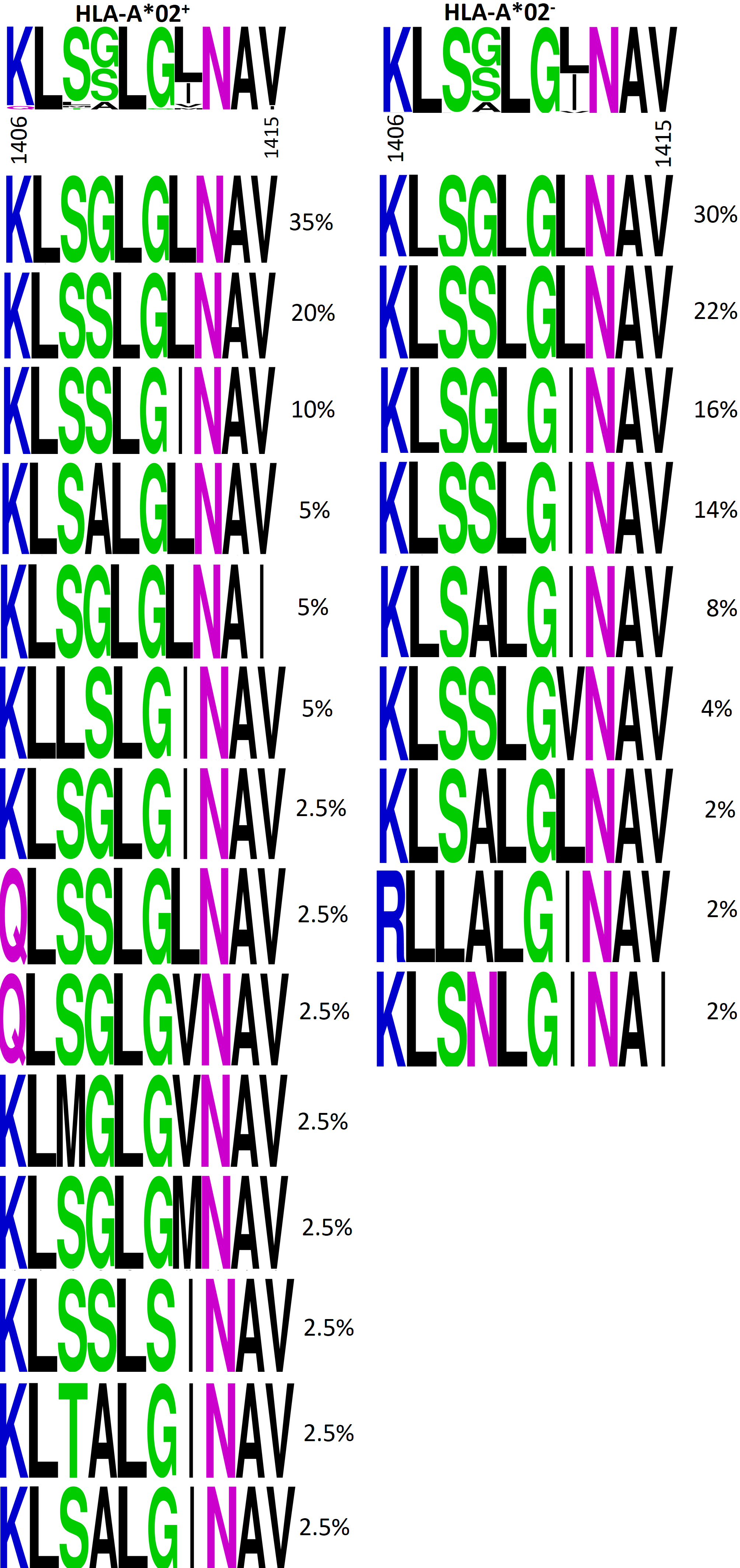

Supplement: Supplementary Figure S2 — Distribution of dominant aminoacid sequences in epitope NS31406 among HLA-A*02-positive and HLA-A*02-negative patients. Amino acids are colored according to their chemical properties: polar amino acids (G, S, T, Y, C, Q, N) are green, basic (K, R, H) blue, acidic (D, E) red and hydrophobic (A, V, L, I, P, W, F, M) amino acids are black. Height of letters within the stack indicates the relative frequency of each amino acid at this position. Sequence logos were generated using WebLogo generator (57). [file Image_2.tif]

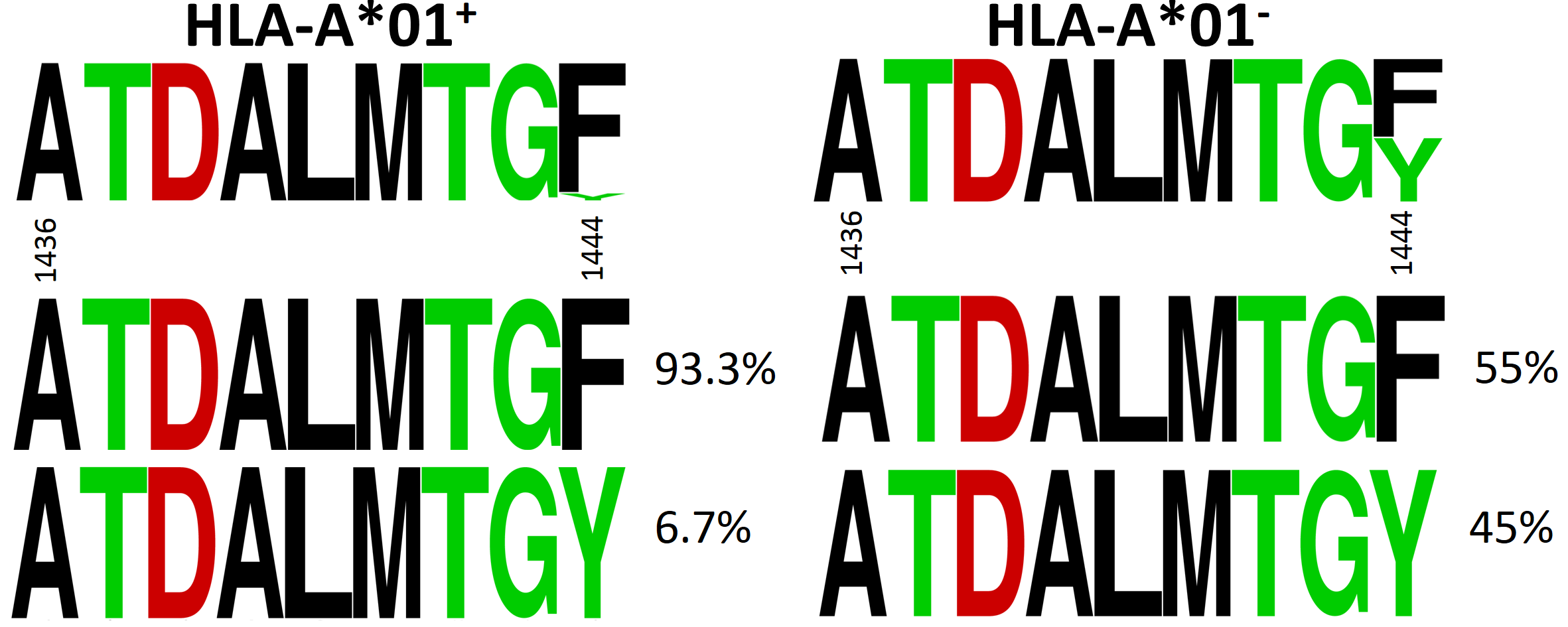

Supplement: Supplementary Figure S3 — Distribution of dominant aminoacid sequences in epitope NS31436 among HLA-A*01-positive and HLA-A*01-negative patients. Amino acids are colored according to their chemical properties: polar amino acids (G, S, T, Y, C, Q, N) are green, basic (K, R, H) blue, acidic (D, E) red and hydrophobic (A, V, L, I, P, W, F, M) amino acids are black. Height of letters within the stack indicates the relative frequency of each amino acid at this position. Sequence logos were generated using WebLogo generator (57). [file Image_3.tif]

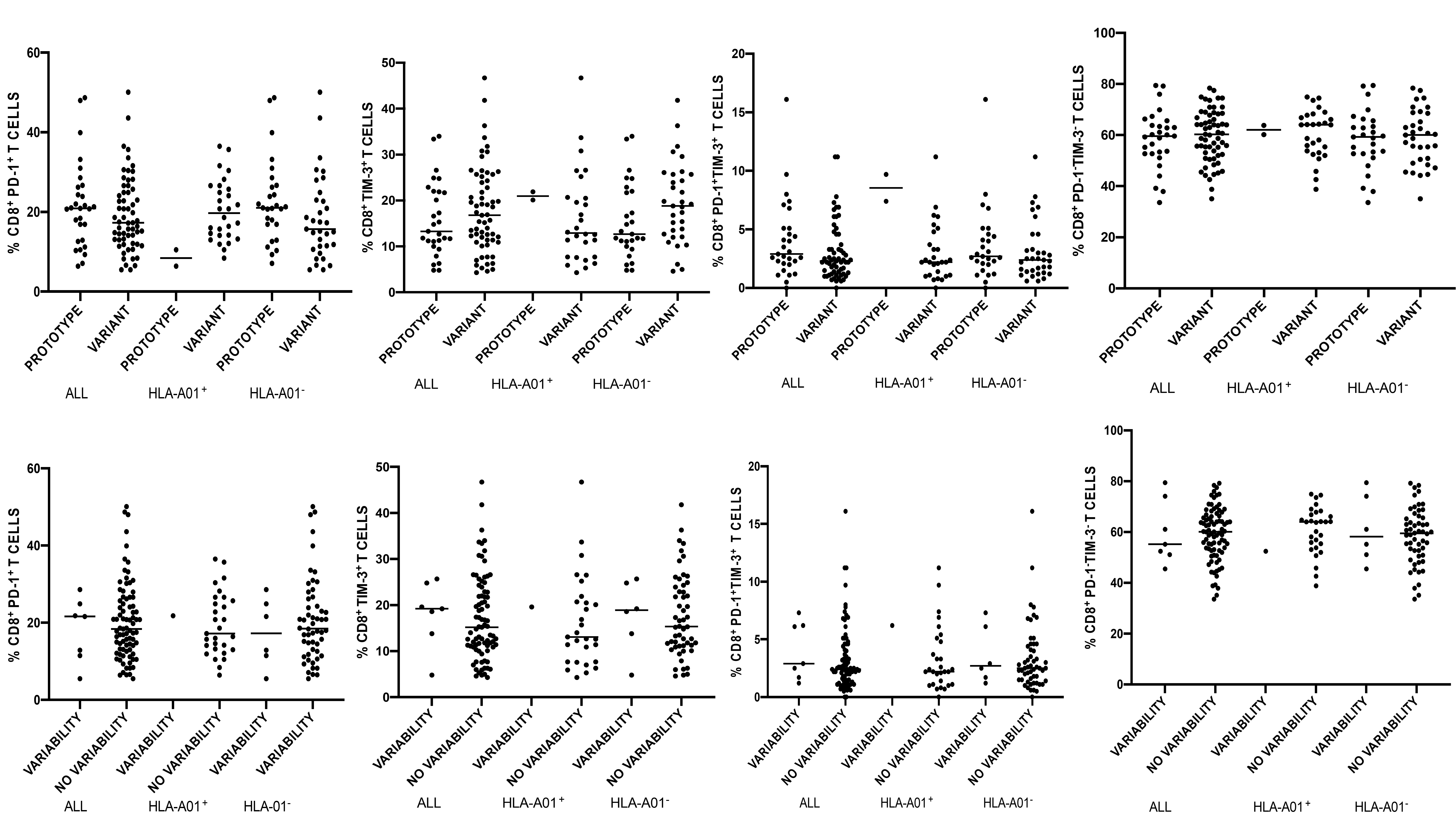

Supplement: Supplementary Figure S4 — Percentages of peripheral blood CD8+ T-cells expressing PD-1/Tim-3 in 90 patients infected with either NS31436 prototype (GenBank EU255962.1) or variant epitope sequence as the dominant strain (upper panel). Lower panel shows intrahost aminoacid variability of this epitope where no variability denotes the presence of a single variant and variability indicates ≥ 2 variant sequences. Horizontal lines represent median values. [file Image_4.tif]

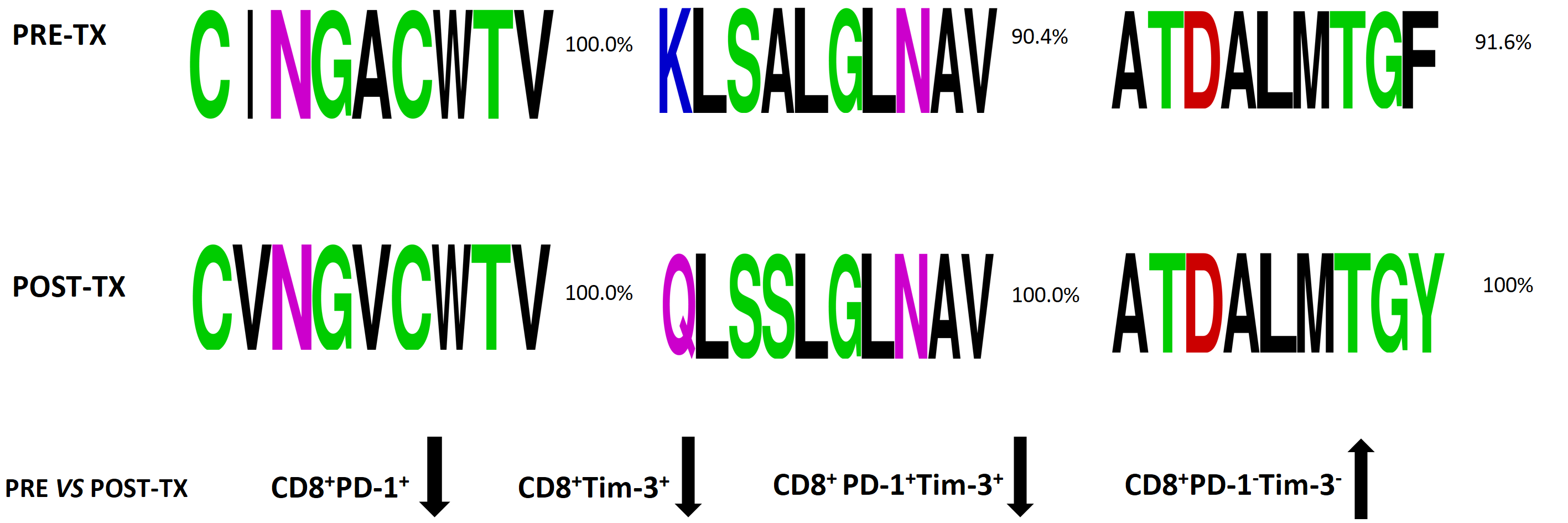

Supplement: Supplementary Figure S5 — Evolution of dominant aminoacid sequences in epitope NS31073, NS31406, NS31436 and percentages of CD8+ T-cells expressing PD-1/Tim-3 in the non-responder to treatment. PRE-TX-before treatment, POST-TX- post-treatment. Amino acids are colored according to their chemical properties: polar amino acids (G, S, T, Y, C, Q, N) are green, basic (K, R, H) blue, acidic (D, E) red and hydrophobic (A, V, L, I, P, W, F, M) amino acids are black. Sequence logos were generated using WebLogo generator (57). [file Image_5.tif]
